# A fluorescently labelled quaternary ammonium compound (NBD-DDA) to study resistance mechanisms in bacteria

**DOI:** 10.3389/fmicb.2022.1023326

**Published:** 2022-11-24

**Authors:** Niclas Nordholt, Kate O'Hara, Ute Resch-Genger, Mark A. T. Blaskovich, Bastian Rühle, Frank Schreiber

**Affiliations:** ^1^Division of Biodeterioration and Reference Organisms (4.1), Department of Materials and the Environment, Federal Institute for Materials Research and Testing (BAM), Berlin, Germany; ^2^Division of Biophotonics (1.2), Department of Analytical Chemistry, Reference Materials, Federal Institute for Materials Research and Testing (BAM), Berlin, Germany; ^3^Centre for Superbug Solutions, Institute for Molecular Bioscience, The University of Queensland, Brisbane, QLD, Australia

**Keywords:** quaternary ammonium compound, QAC, fluorescence, antimicrobial resistance, efflux, TolC

## Abstract

Quaternary ammonium compounds (QACs) are widely used as active agents in disinfectants, antiseptics, and preservatives. Despite being in use since the 1940s, there remain multiple open questions regarding their detailed mode-of-action and the mechanisms, including phenotypic heterogeneity, that can make bacteria less susceptible to QACs. To facilitate studies on resistance mechanisms towards QACs, we synthesized a fluorescent quaternary ammonium compound, namely *N*-dodecyl-*N*,*N*-dimethyl-[2-[(4-nitro-2,1,3-benzoxadiazol-7-yl)amino]ethyl]azanium-iodide (NBD-DDA). NBD-DDA is readily detected by flow cytometry and fluorescence microscopy with standard GFP/FITC-settings, making it suitable for molecular and single-cell studies. As a proof-of-concept, NBD-DDA was then used to investigate resistance mechanisms which can be heterogeneous among individual bacterial cells. Our results reveal that the antimicrobial activity of NBD-DDA against *Escherichia coli*, *Staphylococcus aureus* and *Pseudomonas aeruginosa* is comparable to that of benzalkonium chloride (BAC), a widely used QAC, and benzyl-dimethyl-dodecylammonium chloride (BAC_12_), a mono-constituent BAC with alkyl-chain length of 12 and high structural similarity to NBD-DDA. Characteristic time-kill kinetics and increased tolerance of a BAC tolerant *E. coli* strain against NBD-DDA suggest that the mode of action of NBD-DDA is similar to that of BAC. As revealed by confocal laser scanning microscopy (CLSM), NBD-DDA is preferentially localized to the cell envelope of *E. coli,* which is a primary target of BAC and other QACs. Leveraging these findings and NBD-DDA‘s fluorescent properties, we show that reduced cellular accumulation is responsible for the evolved BAC tolerance in the BAC tolerant *E. coli* strain and that NBD-DDA is subject to efflux mediated by TolC. Overall, NBD-DDA’s antimicrobial activity, its fluorescent properties, and its ease of detection render it a powerful tool to study resistance mechanisms of QACs in bacteria and highlight its potential to gain detailed insights into its mode-of-action.

## Introduction

**Figure 1:**
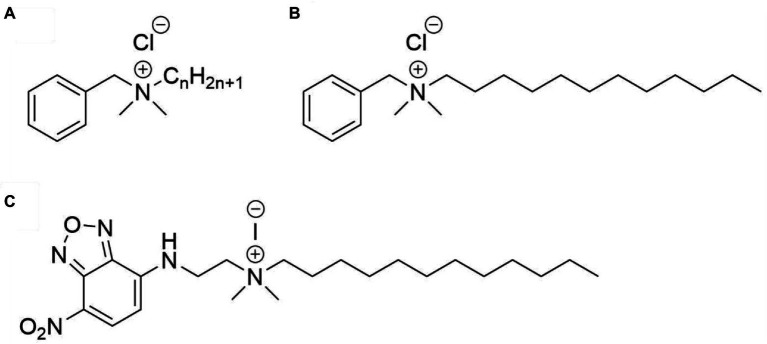
Structures of the compounds used in this study. **(A)** Benzalkonium chloride, with n ranging from 8 to 18, **(B)** benzyl-dimethyl-dodecylammonium chloride (BAC_12_), and **(C)** N-dodecyl-N,N-dimethyl-[2-[(4-nitro-2,1,3-benzoxadiazol-7-yl)amino]ethyl]azanium-iodide (NBD-DDA).

Quaternary ammonium compounds (QACs) were first admitted to the market in the 1940s and are widely used as disinfectants, preservatives, surfactants, and antiseptics (Pereira and Tagkopoulos, 2019). They are positively charged surfactants that possess bactericidal, fungicidal, and viricidal activity (Gilbert and Moore, 2005; Kwaśniewska et al., 2020). QACs are named after a quaternary nitrogen comprising the cationic headgroup, which carries hydrophobic moieties (Figure 1). Among the most widely used QACs is benzalkonium chloride (BAC). Benzalkonium chloride is a cationic surfactant and consists of a mixture of alkyl-benzyl-dimethyl-ammonium chlorides (ADBAC) with alkyl chain lengths varying from 8 to 18 carbon atoms (Figure 1). ADBACs with the alkyl-chain lengths C_12_, C_14_, and C_16_ possess the highest antimicrobial activity (Daoud et al., 1983; Jono et al., 1986) and typically make up the largest mass fraction in BAC (DeLeo et al., 2020).

The main target of BAC, and QACs in general, is thought to be the bacterial cell envelope (Gilbert and Moore, 2005; Barros et al., 2022). The general mode of action likely proceeds as follows: the cationic headgroup attaches to anionic sites on the cell surface, which possesses a net negative charge. Displacement of membrane stabilizing cations (Ca^2+^, Mg^2+^) and integration of the hydrophobic region into the membrane cause membrane instability, changes in membrane fluidity, and leakage of cytoplasmic material (Gilbert and Moore, 2005; Tischer et al., 2012). The mode of action of BAC and other QACs appears to be dose dependent and the detailed mechanisms and their kinetics are still not fully understood (Tischer et al., 2012; Zhang et al., 2015; Inácio et al., 2016; Kwaśniewska et al., 2020).

Resistance mechanisms to QACs, such as BAC, involve changes to the cell envelope, multi-drug efflux, and target degradation, as well as increased biofilm formation (Kampf, 2018; Pereira and Tagkopoulos, 2019). The fact that there are still genes being identified that play a role in the reduced susceptibility to BAC suggests that the exact mechanisms and pathways that reduce the susceptibility to QACs remain underexplored (Nordholt et al., 2021). For many mutations that confer resistance or tolerance to BAC and related compounds, the mechanistic consequence for resistance is not known. For example, do mutations in genes pertaining the cell envelope reduce the rate or amount of QAC adsorbed to the outer membrane? Or do the changes in membrane composition increase membrane stability and, thus, allow bacteria to adsorb larger amounts of QAC before the membrane is compromised? How does efflux contribute to reduced susceptibility? How do QACs interact with components of the periplasm and the cytoplasm? And what are the kinetics of QAC induced death, from adsorption to cell death? Another open question is to which extent phenotypic heterogeneity affects the survival of exposure to QACs, and what are the determinants of individual cells that make them more or less susceptible to QACs.

In this study, we synthesized a fluorescently labelled QAC, NBD-DDA, providing a valuable tool to address open research questions related to resistance mechanisms towards QACs. We characterized its antimicrobial activity against three bacterial species relevant for antimicrobial resistance (AMR), *E. coli*, *S. aureus*, and *P. aeruginosa* and compared it to BAC and benzyl-dimethyl-dodecylammonium chloride (BAC_12_). We demonstrated the potential of NBD-DDA for mechanistic studies by showing that reduced accumulation of NBD-DDA is associated with increased survival in the presence of BAC and that NBD-DDA efflux is mediated by multidrug efflux pumps utilizing TolC. Furthermore, we demonstrated the potential of NBD-DDA for single-cell studies with fluorescence microscopy and flow cytometry. In the future, we anticipate NBD-DDA, and similar compounds that can be synthesized with the presented protocol, to become a powerful tool to advance the knowledge on the mode-of-action and reaction kinetics of QACs and to study the mechanisms that govern the reduced susceptibility of microorganisms to these compounds.

## Materials and methods

### Chemicals for synthesis of NBD-DDA

*N*,*N*-dimethylethylenediamine (Merck, 95%), 4-chloro-7-nitrobenzofurazan (NBD-Cl; Merck, 98%), 1-iodododecane (Merck, 98%), potassium acetate (Th. Geyer, 99%), ethanol (Th. Geyer, absolute, 99.9%), acetone (Th. Geyer, 99.5%), dichloromethane (DCM; Th. Geyer, HPLC-Grade, 99.9%), and methanol (Th. Geyer, 99.8%) were used as received without further purification. Spectroscopic measurements were carried out in spectroscopic grade dimethylsulfoxide (DMSO; Honeywell, absolute, for UV-spectroscopy, 99.8%) and phosphate buffered saline (PBS; 1x, pH = 7.4). PBS was prepared from a pre-weighed tablet (Th. Geyer) with Milli-Q water (> 18 MΩ) according to the instructions of the manufacturer.

### Spectrometry for product characterization

^1^H and ^13^C NMR spectra were recorded on an ECP500 (JEOL; NBD-DMA) or an ECP600 (JEOL; NBD-DDA) at a proton resonance frequency of 500 MHz and 600 MHz, respectively, and calibrated to the residual solvent signals at δ 7.26 (^1^H) and δ 77.16 (^13^C) for chloroform and δ 2.50 (^1^H) and δ 39.52 (^13^C) for DMSO (Fulmer et al., 2010), respectively. Electrospray ionization time-of-flight mass spectrometry (ESI-TOF-MS) measurements were done with an Agilent 6,210 ESI-TOF mass spectrometer (Agilent Technologies). The solvent flow rate was adjusted to 4 μl/min, the spray voltage set to 4 kV, and the drying gas flow rate set to 15 psi (1 bar). All other parameters were adjusted to yield a maximum abundance of the relative [M + H] peak. Fluorescence measurements were done at room temperature using dilute solutions of NBD-DDA in 10 mm x 10 mm quartz cuvettes (Hellma GmbH). Fluorescence emission measurements were performed with a calibrated spectrofluorometer FSP-920 (Edinburgh Photonics), equipped with a Xenon lamp and double monochromators. The fluorescence emission spectra were corrected for the wavelength dependent spectral responsivity of the emission channel (emission correction) and the fluorescence excitation spectra for the wavelength dependent spectral radiant power of the excitation spectra (excitation correction) (Resch-Genger et al., 2005).

### Synthesis of *N*,*N*-dimethyl-N′-(4-nitro-5-benzofurazanyl)-1,2-ethanediamine (NBD-DMA)

NBD-DMA was synthesized following a published procedure with slight modifications (Bednarczyk et al., 2000). In short, potassium acetate (1.083 g, 11 mmol) was added to a solution of *N*,*N*-dimethylethylenediamine (3 ml, 27 mmol) in ethanol (20 ml). NBD-Cl (1.1 g, 5.4 mmol) in ethanol (60 ml) was added slowly to this solution at 45°C and the reaction was stirred overnight. After removing volatiles *in vacuo*, the residue was taken up in water and extracted with ethyl acetate. Here, a much larger volume of solvents than described in the original publication had to be used during the extraction steps because of the strong coloration of both phases. The combined organic extracts were dried over magnesium sulfate and concentrated *in vacuo*. Purification by flash column chromatography on silica gel (70–230 mesh) with methanol:DCM = 6:94 (v/v) yielded the title compound as a brown solid (0.63 g, 2.5 mmol, 47%). ^1^H-NMR (CDCl_3_): δ 2.33 (s, 6H), δ 2.71 (t, 2H), δ 3.48 (m, 2H), δ 6.13 (d, 1H), δ 8.49 (d, 1H); ^13^C-NMR (CDCl_3_): δ 40.6, 45.1, 56.2, 98.9, 136.6, 144.1, 144.4; ESI-TOF-MS m/z: calculated for C_10_H_14_N_5_O_3_ ([M + H]^+^) 252.1091, observed 252.1153.

### Synthesis of *N*-dodecyl-*N*,*N*-dimethyl-[2-[(4-nitro-2,1,3-benzoxadiazol-7-yl)amino]ethyl]azanium-iodide (NBD-DDA)

0.3 g (1 mmol) of NBD-DMA was dissolved in 10 ml of acetone. 1-Iodododecane (0.36 ml, 5.08 mmol) was added, and the mixture was heated at 70°C until the reaction was complete. The amber-yellow ammonium salt was collected by vacuum filtration, washed 2x with acetone (2 × 10 ml), and dried at room temperature, yielding the title compound as an amber-yellow solid (0.246 g, 0.45 mmol, 45%). ^1^H-NMR (DMSO-d6): δ 0.84 (t, 3H), δ 1.24 (bm, 18H), δ 1.65 (m, 2H), δ 3.14 (s, 6H), δ 3.38 (m, 2H), δ 3.66 (t, 2H), δ 3.99 (m, 2H), δ 6.60 (d, 1H), δ 8.58 (d, 1H), δ 9.33 (s, 1H); ^13^C-NMR (DMSO-d6): δ 13.9, 21.8, 22.1, 25.7, 28.5, 28.7, 28.8, 28.9, 29.0, 31.3, 37.2, 50.6, 59.8, 62.1, 63.7, 100.1, 100.2, 122.1137.6, 144.0, 144.5; ESI-TOF-MS m/z: calculated for C_22_H_38_N_5_O_3_ ([M-I]^+^) 420.2969, observed 420.3005.

### Bacterial strains and growth conditions

Bacteria were cultivated in 100 ml Erlenmeyer flasks filled with 10 ml medium at 37°C with agitation at 220 rpm. *E. coli* K-12 MG1655, *S. aureus* SH1000, and *P. aeruginosa* MPAO1 (Varadarajan et al., 2020) were used as reference strains to determine the antimicrobial activity of NBD-DDA. The BAC-tolerant strain *E. coli* S4 originates from an evolution experiment with BAC and *E. coli* K-12 MG1655 as ancestor (Nordholt et al., 2021). The *ΔtolC* mutant has previously been described by Bergmiller et al. (2017). Unless mentioned otherwise, *E. coli* were, as previously described (Nordholt et al., 2021), cultivated in M9 minimal medium containing: 42 mM Na_2_HPO_4_, 22 mM KH_2_PO_4_, 8.5 mM NaCl, 11.3 mM (NH_4_)_2_SO_4_, 1 mM MgSO_4_, 0.1 mM CaCl_2_, 0.2 mM Uracil, 1 μg/ml thiamine, trace elements (25 μM FeCl_3_, 4.95 μM ZnCl_2_, 2.1 μM CoCl_2_, 2 μM Na_2_MoO_4_, 1.7 μM CaCl_2_, 2.5 μM CuCl_2_, 2 μM H_3_BO_3_) and 20 mM glucose. Cultivation and susceptibility testing of *E. coli* was done in M9 to make the results obtained here comparable to the results we recently obtained for BAC (Nordholt et al., 2021). *S. aureus* and *P. aeruginosa* were cultivated in cation adjusted Mueller-Hinton broth 2 (MH2; Sigma-Aldrich, 90922). Plating for enumeration of colony forming units was done on LB agar medium (Lennox formulation; 10 g/l tryptone, 5 g/l yeast extract, 5 g/l NaCl, 15 g/l agar).

### Determination of MIC and MBC values

Stationary phase cultures were diluted to a final cell concentration of 10^7^ cfu/ml in a 96-well plate with wells holding a final reaction volume of 200 μl of fresh medium containing different concentrations of the investigated antimicrobial substance. The tested concentrations of BAC (Sigma-Aldrich, 234427), benzyl-dimethyl-dodecylammonium chloride (BAC_12_; Sigma-Aldrich, 13380) and NBD-DDA were 10, 20, 30, 40, 60, 100, and 150 μM. The plates were incubated at 37°C with agitation for 24 h and growth was assessed visually. To determine the MBC (minimum bactericidal concentration), 10 μl from each well without visible growth were spotted onto LB agar medium and incubated at 37°C for 24 h. The first concentration that reduced the initial cell density by a factor of 10^3^ or more was designated the MBC. NBD-DDA was dissolved in DMSO. To assess the effect of DMSO on growth, a MIC (minimum inhibitory concentration) assay with the solvent DMSO alone was conducted. Growth was not inhibited by DMSO in the concentration range tested here. All experiments were conducted with n = 3 biological replicates and yielded the same MIC and MBC.

### Time-kill assays with *Escherichia coli*

Time-kill assays were conducted as previously described (Nordholt et al., 2021). *E. coli* were inoculated into 10 ml M9 to a defined number of cells (10^4^ cfu/ml) and incubated at 37°C with agitation at 220 rpm. After 24 h, OD_600_ was determined and adjusted to an OD_600_ of 0.01 (~10^7^ cfu/ml) in spent medium in 2 ml tubes (PP, LABSOLUTE) in a total volume of 900 μl. To maintain the cellular physiology in the stationary phase, time-kill assays were conducted in spent medium from the pre-culture, which was obtained by removing cells from the pre-culture medium by centrifugation for 2 min at 16000 g. After dilution of the cells, 10 μl were sampled to determine the initial cell concentration. Antimicrobials were added and cultures were incubated at 37°C with agitation (1,200 rpm) in a tabletop Thermomixer (STARLAB). Samples of 10 μl were taken and immediately serially diluted in 96-well microplates with 90 μl phosphate buffered saline (PBS, pH 7; 10 mM Na_2_HPO_4_, 1.76 mM KH_2_PO_4_, 2.68 mM KCl, 137 mM NaCl) per well. To enumerate colony forming units (cfu), 10 μl from each well were spotted on square LB agar plates. Plates were left to dry and incubated at 30°C for 16–24 h. To determine the fraction of survivors for single time point incubations, the initial time point before addition of antimicrobial and the final time point after 20 min of exposure were sampled.

### Flow cytometry

A stationary phase culture of *E. coli* was diluted 1:200 in PBS which was filtered with a 0.2 μm syringe filter and contained 10 μM of NBD-DDA. Cells were then incubated for different times and subjected to flow cytometry. Flow cytometric measurements were conducted using a Cytoflex S (Beckman-Coulter) with the following settings: flow rate 10 μl/min, primary threshold forward scatter-height (FSC) 1,000, secondary threshold side scatter-height (SSC) 1,000, and 30 s measurement time. These settings allowed the detection of unlabelled cells in the FSC and SSC channels. Between 50,000–70,000 events (i.e., cells) were recorded. NBD-DDA fluorescence was detected in the FITC channel using an excitation wavelength of 488 nm and an emission detection window of 525 ± 20 nm.

### Staining and confocal imaging

One milliliter of a stationary culture of *E. coli* were pelleted by centrifugation and resuspended in PBS. NBD-DDA and the membrane dye SynaptoRed C2 (Sigma-Aldrich, S6689) were added to a final concentration of 10 μM. The suspension was applied to an object slide and covered with a no. 1.5H cover slip (Marienfeld). Imaging was done on a confocal laser scanning microscope Leica SP8 equipped with a white light laser (Superk Extreme EXW-9 NIM, NKT Photonics, Denmark) using a 100x oil immersion objective with a numerical aperture of 1.4. The fluorescence of NBD-DDA was excited at 470 nm (laser power of 34%) and recorded in the spectral window of 500–600 nm. The membrane stain SynaptoRed C2 used for colocalization studies was excited at 570 nm (laser power 24.6%) and detected in the spectral window of 650–800 nm. Both channels were sequentially excited to avoid spectral bleed through. The pixel size of the recorded images was 41.4 nm.

A z-stack with 10 images and a step size of 0.26 μm was recorded. The images were deconvoluted with the software Huygens Essential (Version 17.04, Scientific Volume Imaging B.V., The Netherlands) with default settings and a maximum intensity projection of the deconvoluted z-stacks was created. The intensity histograms of the images were adjusted to improve the visibility of the fluorescence signal.

### Fluorometric NBD-DDA accumulation assay

Stationary phase cultures of *E. coli* were harvested by centrifugation for 4 min at 6,000 g, the supernatant was removed and the OD_600_ was adjusted to 1 (approx. 10^9^ cfu/ml) in PBS and aliquoted into 200 μl samples. NBD-DDA was added to a final concentration of 10 μM and samples were incubated for 45 min. After another centrifugation step (4 min, 6,000 g), the supernatant was removed, cells were resuspended in PBS and the fluorescence was determined with a Qubit 4 fluorometer (Thermo Fisher Scientific, Wilmington, DE, USA) with excitation at 470 nm and emission at 510–580 nm. Cells in PBS without NBD-DDA were used to determine the autofluorescence background, which was subtracted from the fluorescence signal obtained with NBD-DDA. Autofluorescence made up less than 1% of the fluorescence signal of the NBD-DDA treated samples.

### NBD-DDA efflux assays

The methods to determine efflux directly, by following decrease of fluorescence in cells, and indirectly, by measuring accumulation, are based on methods described earlier (Blair and Piddock, n.d.; Coldham et al., 2010). Overnight cultures of *E. coli ΔtolC* (Bergmiller et al., 2017) and the corresponding wild-type were diluted 1:100 in fresh LB and grown to an OD_600_ of 0.2–0.4. After removal of the growth medium by centrifugation for 4 min at 6000 g, the OD was adjusted to OD 0.4 in PBS containing 10 μM NBD-DDA and incubated for 60 min at 37°C with shaking to load the cells with NBD-DDA. To determine accumulation of NBD-DDA, an aliquot was taken, diluted 1:200 in PBS and subjected to flow cytometry. Cells were then harvested by centrifugation for 4 min at 6000 g, resuspended in fresh LB and incubated at 37°C with shaking for 60 min. An aliquot was again taken, diluted 1:200 in PBS and subjected to flow cytometry to determine the fluorescence reduction rate related to efflux. In addition, after removal of the cells from the sample by centrifugation, the supernatant was measured in a Qubit 4 fluorometer (ThermoFisher) to determine NBD-DDA effluxed by the cells. Additionally, the pelleted cells were resuspended in PBS and subjected to confocal imaging with settings as described above to visualize remaining NBD-DDA levels inside the cytoplasm and the membrane. Single z-slice images were recorded. Prior to quantification, individual images were background corrected by subtracting the average pixel intensity of an area without cells from all pixel intensities. Average pixel intensities inside of cell contours (cytoplasm) and on the contours (cell envelope) were quantified with FIJI distribution of ImageJ (Schindelin et al., 2012).

## Results and discussion

### Synthesis of NBD-DDA and chemical characterization

Quaternary ammonium compounds (QACs) are a class of surfactants that contain a quaternary nitrogen and a hydrophobic region (Gilbert and Moore, 2005). Generally, their main mode-of-action involves attachment of the cationic quaternary nitrogen to the cell envelope, followed by destabilization of the membrane through insertion of the hydrophobic region (Gilbert and Moore, 2005). Perhaps the most prominent example of QACs are benzalkonium chlorides (BAC). Benzalkonium chlorides (*N*-alkyl-*N*-benzyl-*N*,*N*-dimethylammonium chlorides) are a class of compounds with the general structure depicted in Figure 1A. When employed as biocides, often a mixture of BAC with different alkyl chain lengths are used, with compounds with C_12_, C_14_, and C_16_ chains possessing the highest antimicrobial activity (Daoud et al., 1983; Jono et al., 1986) and typically making up the largest mass fraction in BAC biocides (DeLeo et al., 2020). Fluorophore-labeled QACs such as NBD-DDA (Figure 1C) are structurally very similar and can be readily synthesized in two steps from commercially available precursors. In the first step, *N*,*N*-dimethylethylenediamine is reacted with NBD-chloride in a nucleophilic substitution (S_N_) reaction, yielding the intermediate product *N*,*N*-dimethyl-*N*′-(4-nitro-5-benzofurazanyl)-1,2-ethanediamine (NBD-DMA) (Bednarczyk et al., 2000). This can then be reacted with alkyl halides (especially alkyl iodides) of various chain lengths in another S_N_ reaction, yielding the final NBD labeled quaternary ammonium compound.

In this study, we chose dodecyl iodide for the functionalization in the second step to obtain a derivative (NBD-DDA) with a C_12_ alkyl-chain length, that functionally mimics one of the most active compounds typically found in BAC mixtures (Figures 1B,C; Daoud et al., 1983; Jono et al., 1986; Pereira and Tagkopoulos, 2019). Importantly, compounds similar to NBD-DDA with varying alkyl-chain lengths mimicking QAC components other than the C_12_ derivative or even using benzyl or aryl groups instead of alkyl groups can be synthesized with minor modifications to the synthesis protocol. This can pave the way for mechanistic studies of the antimicrobial activity of different alkyl-chain lengths in BACs, and other QACs, which can vary between bacterial species (Daoud et al., 1983; Jono et al., 1986).

The dye NBD was chosen due to its small size, its structural similarity to a simple aromatic substituent and its fluorescence emission in the visible spectrum, which can be excited with typical laser or light emitting diode (LED) light sources around 488 nm. Another advantage of NBD is the insensitivity of its optical properties towards changes in chloride concentration and pH in the physiologically relevant range of pH 5.0–7.4. Moreover, NBD dyes are popular lipid membrane probes owing to the ability of their derivatives to be readily incorporated into lipid membranes and the sensitivity of their optical properties to the polarity of the dye microenvironment (Chattopadhyay, 1990; Bednarczyk et al., 2000; Amaro et al., 2016).

The chemical structure, polarity, and steric demand of NBD-DDA (bounding box length [A]: 25, width [A]: 12, height [A]: 7, volume [A^3^]: 2100) and BAC12 (bounding box length [A]: 21, width [A]: 10, height [A]: 7, volume [A^3^]: 1470) as determined after universal force field minimization are quite similar, making NBD-DDA a well-suited analogue for benzyl-dimethyl-dodecylammonium chloride (Figure 1) show comparable properties. Overall, NBD-DDA is structurally very similar to benzyl-dimethyl-dodecylammoniumchlorid based on steric demand and polarity (Figure 1).

**Figure 2:**
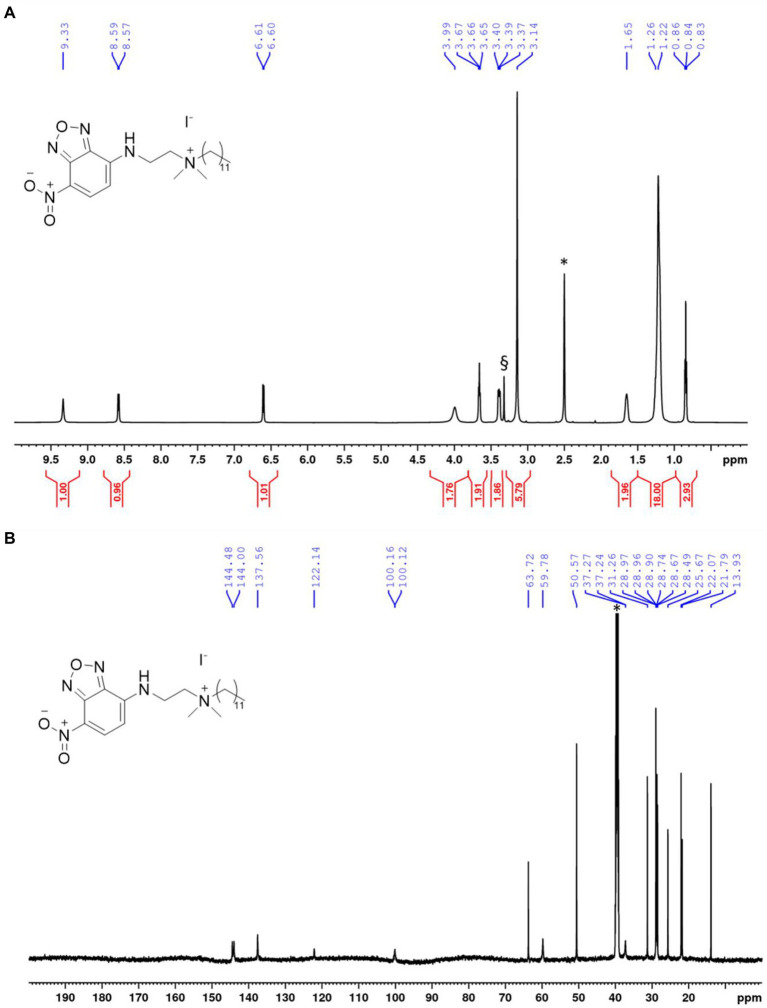
NMR spectra of NBD-DDA. **(A)** ^1^H-NMR of NBD-DDA. * and § denote residual solvent peaks from DMSO and H_2_O, respectively. **(B)** ^13^C-NMR of NBD-DDA. * denotes the residual solvent peak from DMSO.

**Figure 3:**
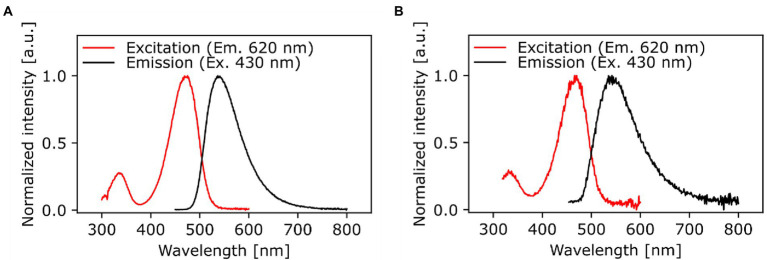
Spectrally corrected normalized fluorescence excitation and emission spectra of NBD-DDA in **(A)** DMSO and **(B)** PBS (1x, pH 7.4). The excitation spectra were monitored at an emission wavelength of 620 nm and the emission spectra were excited at 430 nm.

The chemical structure and purity of NBD-DDA was confirmed by high resolution mass spectrometry (HR-MS) and NMR spectroscopy (Figure 2). Subsequently, the absorption and emission properties of NBD-DDA were determined to clarify whether the standard FITC settings could be used for the fluorescence microscopy and flow cytometry studies. The optical characterization of the compound by fluorescence spectroscopy (Figure 3) in DMSO and PBS showed two absorption bands at 335 nm and 468 nm respectively, with the strongest absorption band being located at 468 nm. The fluorescence emission spectra show a strong emission band with a maximum at 537 nm in DMSO and a weaker fluorescence band at 542 nm in PBS. These excitation and emission characteristics are very similar to GFP and FITC, making this dye well suited for fluorescence microscopy imaging studies using the same settings as applied for GFP and FITC.

### Antimicrobial activity of NBD-DDA is comparable to BAC

The antimicrobial activity of NBD-DDA against *E. coli*, *S. aureus*, and *P. aeruginosa* was assessed and compared to that of BAC and benzyl-dimethyl-dodecylammonium chloride (BAC_12_), which possesses the same alkyl chain length as NBD-DDA (Figure 1). To this end, the minimum inhibitory concentration (MIC) and the minimum bactericidal concentration (MBC) were determined. The MIC is the concentration which inhibits growth within 24 h and the MBC is the concentration which reduces the number of cfu by a factor 10^3^ or more, i.e., kills ≥99.9% of the cells, within 24 h. Additionally, a BAC tolerant *E. coli* strain (*E. coli* S4) was included in the assays to test whether NBD-DDA and BAC share a similar mode of action. The strain *E. coli* S4 was isolated from a laboratory evolution experiment and shows increased short-term survival in the presence of BAC, without changes in the MIC or MBC (Nordholt et al., 2021). The MIC and MBC of all substances tested for *P. aeruginosa* were above 150 μM, the highest concentration in the assay. The MIC and MBC of NBD-DDA for all other tested strains were comparable to the MIC and MBC of BAC and BAC_12_ (Table 1). These results indicate that the fluorescent NBD moiety does not affect the antimicrobial properties of NBD-DDA as determined by MIC and MBC.

**Table 1:** MIC and MBC values of NBD-DDA as compared to those of BAC and BAC_12_ for different bacterial strains.

**Strain**	MIC*			MBC*			**Medium**
	**NBD-DDA**	**BAC**_**12**_	**BAC**	**NBD-DDA**	**BAC**_**12**_	**BAC**	
*E. coli*	20	30	20	20	30	20	M9
*E. coli S4*	20	30	20	20	30	20	M9
*P. aeruginosa*	>150	>150	>150	>150	>150	>150	MH2
*S. aureus*	10	10	20	30	30	20	MH2

*Tested concentrations: 10, 20, 30, 40, 60, 100, and 150 μM. Experiments were conducted with *n* = 3 biological replicates and yielded the same MICs and MBCs.

### Common mode of action of BAC and NBD-DDA

**Figure 4:**
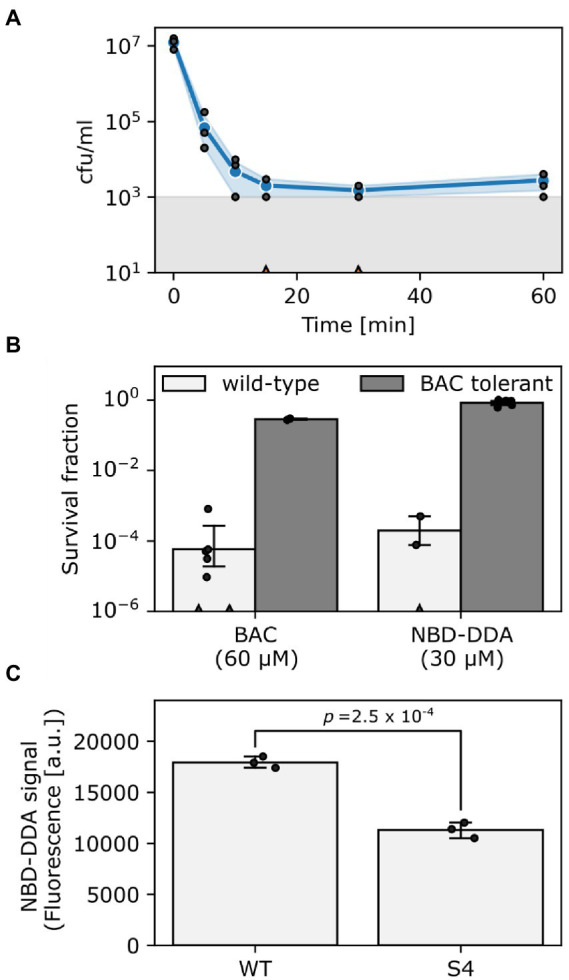
NBD-DDA and BAC share a similar mode-of-action. **(A)** Time-kill curve of *E. coli* wild-type exposed to 30 μM NBD-DDA. **(B)** Survival of *E. coli* wild-type and the BAC tolerant *E. coli* S4 after 20 min of treatment with BAC or NBD-DDA. **(C)** NBD-DDA fluorescence is reduced in the BAC tolerant strain *E. coli* S4, suggesting reduced binding as underlying tolerance mechanism. The *value of p* in panel C was obtained by a two-tailed, unpaired t-test of the fluorescence values. Blue lines and circles in panel A and bars in panels B and C show the geometric mean. Black dots indicate individual experiments. Errors are 95% confidence intervals of the geometric mean obtained by bootstrapping. The grey area in panel A shows the detection limit. Experiments with zero-counts in panels A and B are indicated by triangles on the x-axis and were omitted.

In a recent study, we showed that *E. coli* populations display heterogeneity in their ability to survive lethal doses of BAC, as was apparent from bimodal killing kinetics (Nordholt et al., 2021). Here, we reasoned that if NBD-DDA has a similar mode-of-action as BAC, *E. coli* should also display bimodal killing kinetics. Indeed, this was the case when *E. coli* was exposed to a lethal concentration of NBD-DDA (Figure 4A). These results were further corroborated by the fact that the BAC-tolerant *E. coli* strain S4 showed an increased short-term survival in the presence of NBD-DDA in comparison to the wild type (Figure 4B). The time-kill experiments with NBD-DDA and BAC suggested a slight increase in potency of NBD-DDA as compared to BAC because killing with 30 μM NBD-DDA resulted in a similar reduction in cfu as compared to 60 μM BAC after 20 min of exposure (Figure 4B). Thus, while the MIC and the MBC of NBD-DDA were comparable to those of BAC and BAC_12_ (Table 1), high resolution time-kill curves were able to reveal nuanced differences in the bactericidal activity of BAC and NBD-DDA during short-term exposure.

We previously showed that a reduction in the net negative surface charge contributes to BAC tolerance in *E. coli* S4, presumably decreasing BAC adsorption to the cells (Nordholt et al., 2021). We corroborated these findings by utilizing the fluorescent properties of NBD-DDA to directly measure binding of NBD-DDA by the BAC susceptible wild-type and the BAC tolerant strain S4 in a fluorometer. Briefly, cells were incubated with NBD-DDA, and, after removing the supernatant containing NBD-DDA by centrifugation, fluorescence of the cells was determined fluorometrically. Less NBD-DDA was bound by the BAC tolerant strain (Figure 4C). Due to its fluorescence, the accumulation of the antimicrobial NBD-DDA itself in the cells can be followed, preventing the need for an auxiliary fluorescent probe. Together with our previous findings (Nordholt et al., 2021), the results underscore that an altered cell surface charge contributes to the BAC tolerance in *E. coli* S4 by reducing cellular binding. In future studies NBD-DDA could be utilized to characterize the basis of the susceptibility of other species to QACs. For instance, the question could be addressed as to whether the variation in susceptibilities between different bacterial species (Table 1) can be attributed to differences in the accumulation.

Taken together, our experiments indicate that the biological activity of NBD-DDA is very similar to that of BAC, not only in terms of its efficacy, but also in terms of mode-of-action. Furthermore, we showed that the tolerance against NBD-DDA, and BAC, is mediated through reduced binding by the tolerant *E. coli* strain S4.

### Fluorescence of NBD-DDA facilitates studies on the single-cell level

**Figure 5:**
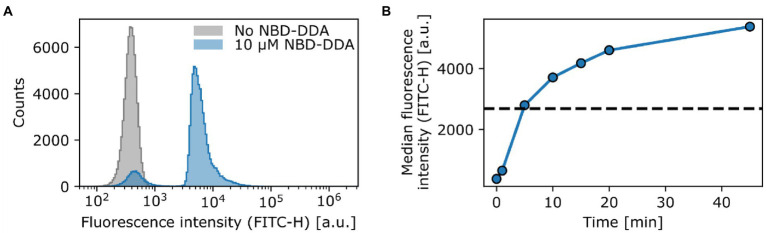
NBD-DDA effectively stains *E. coli* cells and can be detected with flow cytometry. **(A)** Staining of *E. coli* wild-type with a sub inhibitory level of NBD-DDA. **(B)** Staining with NBD-DDA increases with time, with the half maximal saturation of staining being reached after approx. 5 min (dashed line). The data points show the median fluorescence values of 50,000–70,000 cells. The fluorescence was recorded in the FITC channel (excitation at 488 nm; emission detection window of 525 ± 20 nm).). Given are the maximum intensity (height) values of the fluorescence signals.

To demonstrate the potential of NBD-DDA for studying QAC activity on the single-cell level, we stained the *E. coli* wild-type with NBD-DDA. To this end, the cells were incubated with a sub-inhibitory concentration of NBD-DDA (10 μM, 0.5 × MIC) and subsequently assessed by flow cytometry and confocal microscopy. *E. coli* cells stained with NBD-DDA were readily detected in the standard FITC/GFP channel (excitation: 488 nm, emission: 525 ± 20 nm) in the flow cytometer (Figure 5). The cells showed a fluorescence distribution, signifying heterogeneity between individual cells (Figure 5A). After 5 min of incubation, the population of fluorescently stained cells could be already clearly distinguished from the autofluorescence signal of unstained cells (Figure 5A). A small fraction of around 9% of the cells incubated with NBD-DDA did not display a fluorescence signal different from that of the unlabeled cells (Figure 5A). Increasing the staining duration increased the fluorescence signal derived from the bacteria cells (Figure 5B), with half-maximal saturation of staining being reached after 5 min of incubation. The fraction of non-fluorescent cells was invariant to incubation times (data not shown). A possible explanation for the fraction of non-fluorescent cells might be the presence of a persister subpopulation with increased tolerance to QACs through reduced cellular accumulation, for example through changes in the cell surface charge or increased efflux. This would be in line with recent findings that showed that *E. coli* exhibits persistence to BAC, that reduced cell surface charge underlies evolved tolerance against BAC (Nordholt et al., 2021), and that persister cells can show increased efflux activity (Pu et al., 2016).

**Figure 6:**
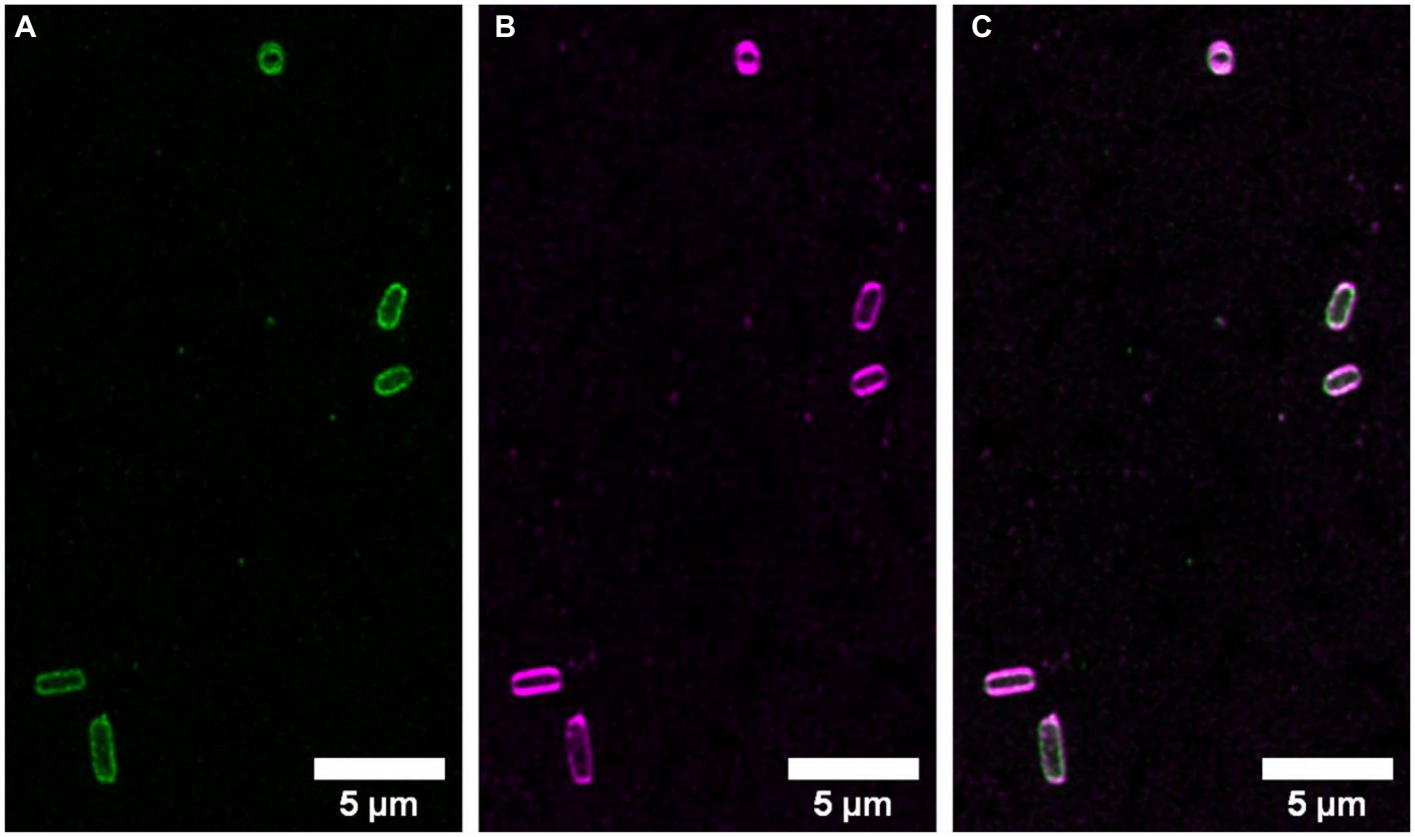
NBD-DDA is localized to the cell envelope of *E. coli*. *E. coli* cells stained with **(A)** 10 μM NBD-DDA and **(B)** with the membrane stain SynaptoRed C2 (10 μM). **(C)** The overlay of both signals shows their colocalization. Shown are the maximum intensity projections of z-stacks with 10 slices. Image histograms were adjusted for visibility.

*E. coli* cells stained with NBD-DDA were also examined using confocal microscopy (Figure 6A). Fluorescence intensity was highest at the cell edges, suggesting that NBD-DDA localizes to the cell envelope. This observation was confirmed by co-staining of the cells with the membrane stain SynaptoRed C2. The signals from both fluorophores revealed dye colocalization (Figures 6B,C). This is in accordance with the assumption that the cell membrane is the primary target of BAC (Gilbert and Moore, 2005; Kwaśniewska et al., 2020). Thus, NBD-DDA could be leveraged in live cell imaging applications of mammalian and bacterial cells to label cell membranes (Deng et al., 2021). For absolute quantification of NBD-DDA, additional experiments are needed to determine the effect of polarity of different cellular microenvironments on the fluorescence signal of NBD-DDA.

The potential of fluorescent probes derived from antimicrobial substances to study drug susceptibility in single bacterial cells has recently been demonstrated for a range of antibiotics (Stone et al., 2019; Łapińska et al., 2022). Here we demonstrate that this approach is also promising for QACs, which are used as disinfectants, antiseptics, and preservatives (Pereira and Tagkopoulos, 2019). We used a sub-inhibitory concentration of NBD-DDA for staining purposes. In future studies, it will be interesting to observe the effects of higher concentrations on the localization within cells and cell consortia such as biofilms, as well as observing the dynamics of NBD-DDA adsorption and killing in individual cells, using time lapse microscopy in a microfluidic device.

### TolC activity is important for QAC efflux

**Figure 7:**
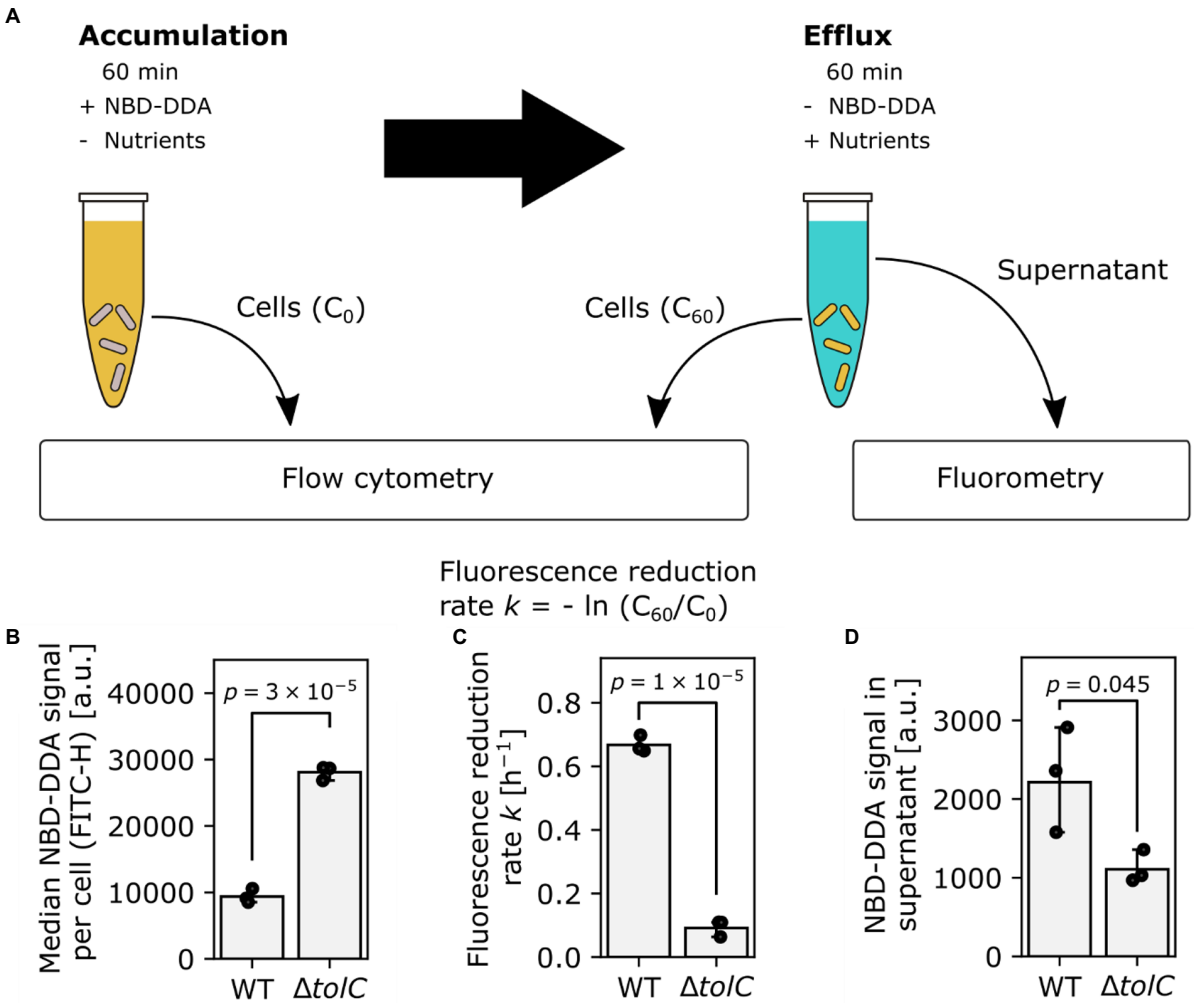
TolC is involved in efflux of NBD-DDA. **(A)** Workflow to determine efflux activity in bacteria, using NBD-DDA. **(B)** Increased median accumulation of NBD-DDA per cell in *E. coli ΔtolC* as determined by flow cytometry suggests decreased efflux activity. **(C)** Reduced fluorescence reduction rate per cell and **(D)** NBD-DDA signal in the supernatant after 60 min are lower in *E. coli ΔtolC*, due to decreased efflux activity. *p*-values were obtained from unpaired, two-tailed t-tests of the fluorescence values or rates. Bars indicate mean of *n* = 3 replicates. Black dots indicate individual experiments. Errors are 95% confidence intervals of the mean obtained by bootstrapping.

After we established the use of NBD-DDA with flow cytometry and confocal laser scanning microscopy, we asked whether NBD-DDA in combination with these techniques can be used to study and quantify efflux as a resistance mechanism to QACs. The outer membrane channel TolC is an important component of various multidrug efflux systems in *E. coli* (Piddock, 2006). Overexpression of *tolC* and its associated efflux pumps have been shown to decrease susceptibility to antimicrobials, including antibiotics and QACs (Buffet-Bataillon et al., 2012; Bergmiller et al., 2017). Conversely, deletion of *tolC* increases the susceptibility to BAC (Nordholt et al., 2021). Here, we utilized NBD-DDA’s fluorescent properties to directly show that deletion of *tolC* results in higher levels of NBD-DDA in the mutant cells as compared to the wild-type due to decreased efflux activity, *via* multiple lines of evidence (Figure 7A). The *ΔtolC* mutant showed increased levels of fluorescence per cell after being incubated with NBD-DDA for 60 min (Figure 7B), suggesting that steady state accumulation is higher due to decreased efflux activity in *ΔtolC*. Furthermore, after removal of NBD-DDA from the solution, the relative fluorescence levels decreased faster in the wild type cells (Figure 7C), indicating decreased efflux of NBD-DDA in the *ΔtolC* mutant. Finally, the NBD-DDA effluxed by the cells into the supernatant after 60 min as determined by fluorometry was significantly higher in the wild type than in the *ΔtolC* mutant (Figure 7D).

**Figure 8:**
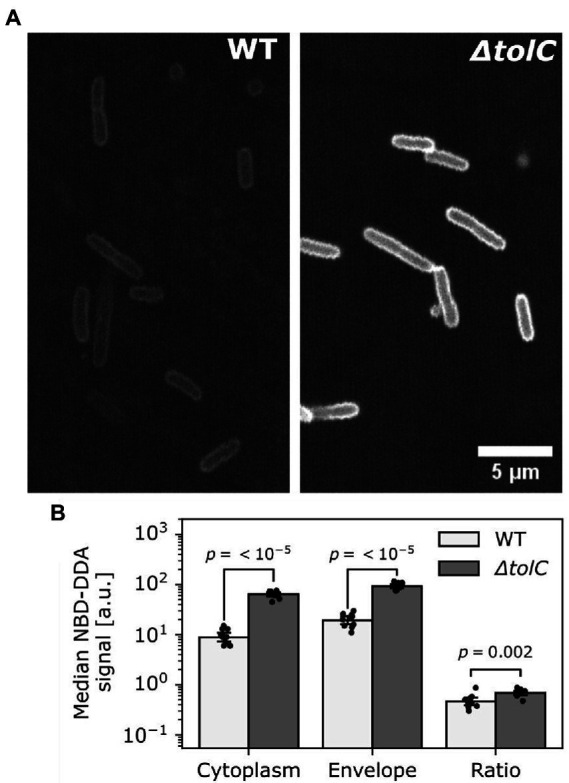
TolC mediated efflux reduced NBD-DDA levels in the cell envelope and the cytoplasm. **(A)** Confocal laser scanning images of *E. coli* wild-type (WT) and *ΔtolC.* Shown are single z-slice raw images recorded with identical settings between strains. **(B)** Quantification of fluorescence pixel intensities within cell contours (cytoplasm) and on the cell contours (envelope), as well as the ratio of cytoplasm over envelope intensity. p-values were obtained from unpaired, two-tailed t-tests of the median pixel intensities of at least 10 pixels. Bars indicate mean of *N* = 10 cells. Black dots indicate individual cells. Errors are 95% confidence intervals of the mean obtained by bootstrapping.

We further asked whether NBD-DDA in combination with confocal microscopy can be used to quantify subcellular localization and how it is affected in the efflux deficient *ΔtolC* mutant. To this end, the cells from the efflux assay were imaged and the fluorescence intensity in the cytoplasm and in the cell envelope was quantified (Figure 8). Our results suggest that TolC activity significantly reduces the accumulation of NBD-DDA in both, the cytoplasm and the cell envelope (Figures 8A,B). Moreover, deletion of TolC results in a higher ratio of cytoplasmic NBD-DDA signal to NBD-DDA signal in the cell envelope (Figure 8B). This suggests that the higher level of NBD-DDA in the cell envelope in *ΔtolC* allows for a higher equilibrium concentration of NBD-DDA in the cytoplasm, where it can potentially interfere with intracellular processes.

Taken together, our results demonstrate that NBD-DDA is a powerful tool to quantitatively assess efflux activity of QACs in bacteria, using various single-cell and bulk methods. In the future, NBD-DDA could be leveraged in sub-cellular localization studies in combination with super-resolution microscopy.

## Conclusion and outlook

We synthesized a fluorescent QAC, NBD-DDA, that is readily detectable by fluorescence methods such as flow cytometry and fluorescence microscopy using standard FITC/GFP settings. We also demonstrated that it can be utilized to study resistance mechanisms of bacteria against QACs, as well as phenotypic heterogeneity on the single-cell level. NBD-DDA has three important properties:

- the antimicrobial activity of NBD-DDA is comparable to that of BAC,
- the mode of action of NBD-DDA is apparently similar to that of BAC,
- the fluorescent properties of NBD-DDA make it suitable to study phenotypic heterogeneity, efflux, cellular accumulation kinetics and subcellular localization.

Leveraging these properties, we were able to gain four insights towards mechanisms of resistance to QACs:

- tolerance to NBD-DDA/BAC in *E. coli* is associated to reduced cellular accumulation in a mutant with reduced surface charge
- accumulation of NBD-DDA/BAC by cells is heterogeneous within isogenic *E. coli* populations
- NBD-DDA/BAC localizes to the bacterial cell envelope
- efflux of NBD-DDA is mediated by TolC and affects accumulation in the cell envelope and the cytoplasm

Our study highlights the potential and versatility of fluorescent QAC analogues, such as NBD-DDA. We anticipate that such compounds will facilitate the understanding of mechanisms related to disinfection, disinfection insusceptibility on the single-cell level and within biofilms, and cytotoxicity of disinfectants. For example, NBD-DDA could be used to:

- determine the mode-of-action of QACs against microorganisms.
- investigate the role of phenotypic heterogeneity on disinfection tolerance, e.g., with respect to tolerant persister subpopulations.
- determine the fate of QACs in biofilms to understand the basis of their recalcitrance to QACs (Lineback et al., 2018; Pereira and Tagkopoulos, 2019).
- identify efflux pumps that are involved in removing QACs from the cell.
- explore the mechanisms which underlie the geno-and cytotoxic effects of QACs in mammalian cells (De Saint Jean et al., 1999; Ferk et al., 2007; Zhang et al., 2015; Datta et al., 2017; Goldstein et al., 2022). Unraveling these mechanisms could guide appropriate counter measures to these effects in consumer products, such as ophthalmic solutions.
- investigate the distribution and fate of QACs when applied to surfaces and in environmental settings (Wood et al., 2021).

## Data availability statement

The original contributions presented in the study are included in the article/supplementary material, further inquiries can be directed to the corresponding author.

## Author contributions

NN conceptualization, data curation, formal analysis, investigation, methodology, software, validation, visualization, writing – original draft preparation, writing – review and editing. KO'H formal analysis, funding acquisition, investigation, writing – review and editing. UR-G resources, writing – review and editing. MB conceptualization, writing – review and editing. BR conceptualization, data curation, formal analysis, investigation, methodology, supervision, validation, visualization, writing – original draft preparation, writing – review and editing. FS conceptualization, formal analysis, funding acquisition, investigation, methodology, project administration, resources, supervision, visualization, writing – original draft preparation, writing – review and editing. All authors contributed to the article and approved the submitted version.

## Funding

The authors acknowledge funding of the work by the Federal Institute for Materials Research and Testing (BAM). The work of KO'H at BAM was performed as part of a traineeship funded by the European Union within the ERASMUS+ program.

## Conflict of interest

The authors declare that the research was conducted in the absence of any commercial or financial relationships that could be construed as a potential conflict of interest.

## Publisher’s note

All claims expressed in this article are solely those of the authors and do not necessarily represent those of their affiliated organizations, or those of the publisher, the editors and the reviewers. Any product that may be evaluated in this article, or claim that may be made by its manufacturer, is not guaranteed or endorsed by the publisher.
